# Imaging features and radiologic-pathologic correlations of inflammatory pseudotumor-like follicular dendritic cell sarcoma

**DOI:** 10.1186/s12880-021-00584-6

**Published:** 2021-03-17

**Authors:** Lingbin Xu, Rong Ge, Shanshan Gao

**Affiliations:** 1 Department of Radiology, Ningbo Medical Center Lihuili Hospital, Ningbo University, 1111 Jiangnan Road, Ningbo, Zhejiang 315000 People’s Republic of China; 2 Department of Diagnosis, Ningbo Diagnostic Pathology Center, Ningbo, Zhejiang 315021 People’s Republic of China; 3 Department of Ultrasound, Hwa Mei Hospital, University of Chinese Academy of Sciences (Ningbo No. 2 Hospital), 41 Northwest Street, Ningbo, Zhejiang 315010 People’s Republic of China

**Keywords:** Inflammatory pseudotumor-like follicular dendritic cell sarcoma, Computed tomography, Histopathology, Magnetic resonance imaging, Ultrasonography

## Abstract

**Background:**

Inflammatory pseudotumor-like follicular dendritic cell sarcoma (IPT-like FDCS) is a rare tumor. This study aimed to reveal the radiological characteristics of IPT-like FDCS by radiologic-pathologic correlation.

**Results:**

We analyzed two cases of IPT-like FDCS in the liver, nine in the spleen, and two in both the liver and spleen concomitantly. IPT-like FDCS presented as well-defined iso- or hypodense masses on unenhanced computed tomography (CT) images in both the liver and spleen. Hyperintensities on T1-weighted images and hypointensities on T2-weighted images with hypointense rings were characteristic features in splenic cases. “Halo signs” were observed in two out of three liver tumors. Hepatic lesions showed significant enhancement, whereas splenic lesions showed only mild enhancement. Delayed annular enhancement was observed in both liver and spleen cases. On ultrasonographic examination, IPT-like FDCS presented as hypoechoic lesions with enhancement similar to that observed on CT. Hyaline fibrous pseudocapsules, which correlated with the hypointensities on T2-weighted images, were microscopically observed at the tumor edge. IPT-like FDCS was characterized by an abundance of small blood vessels and capillaries. Capillaries were also found in the fibrous capsule of some IPT-like FDCSs, which may explain the delayed annular enhancement.

**Conclusions:**

The manifestations of IPT-like FDCS in the liver and spleen showed differences that warrant them to be approached differently during diagnosis. Characteristic radiological findings of IPT-like FDCS included different enhancement patterns between liver and spleen tumors and rim-like hypointensities on T2-weighted images, as well as annular enhancement on CT and magnetic resonance images. These imaging features correlated with tumor pathology.

## Background

Follicular dendritic cell sarcoma (FDCS) is an extremely rare type of tumor. While it usually arises from dendritic cells in lymph nodes, a wide variety of extranodal sites may also be involved. There have been no more than 100 reported cases of FDCS in the literature, mostly case reports, especially of extranodal forms [1–3]. The improved lymphoid hematopoietic classification system was published in 2001, and with the popularization of FDCS-specific immune markers, such as CD21 and CD35, FDCS has been further recognized and reported [4, 5]. Inflammatory pseudotumor-like FDCS (IPT-like FDCS) is a particular subtype of FDCS that develops especially in the liver and spleen. Compared to classical FDCS, IPT-like FDCS is less common [6, 7], with a different histologic appearance and behavior [8]. Due to the inertia of IPT-like FDCS, surgical resection can help to achieve good long-term results [8]. Therefore, an accurate diagnosis prior to a surgery is particularly important. To the best of our knowledge, no comprehensive imaging analysis of IPT-like FDCS exists, especially analysis of radiological-pathological correlations. The purpose of our study was to describe computed tomography (CT), ultrasound, and magnetic resonance imaging (MRI) findings of IPT-like FDCS and relate them to their respective tumor pathological findings, to contribute to better the understanding this uncommon tumor.

## Methods

### Study population

This study was approved by the Institutional Review Board of Ningbo Medical Center Lihuili Hospital, and the study complied with the guidelines of the Healthcare Insurance Portability and Accountability Act. The requirement for written informed consent was waived because of the retrospective study design.

All IPT-like FDCS cases from 2010 to 2019 were retrieved from multiple centers and were confirmed by the local pathological center. In total, 13 patients were included in this retrospective study. The following clinical and pathological data were collected: age, sex, anatomic tumor location, tumor size, clinical symptoms, surgical and pathological findings, treatment outcomes, and follow-up time. For all patients, preoperative images were reviewed.

### Examination methods

Contrast-enhanced MRI was performed in 11 cases, contrast-enhanced CT was performed in eight cases, and both contrast-enhanced MRI and CT were performed in six cases. Ultrasound was performed in four cases, of which one case was also examined by contrast-enhanced ultrasound imaging. Contrast-enhanced CT was performed using a 256-row CT (Revolution; GE Healthcare, Milwaukee, WI) or 16-row multi-detector computed tomography (Brilliance 16; Philips, Eindhoven, The Netherlands). For multiphasic contrast-enhanced CT scanning, an 80–100-mL intravenous iopromide bolus (Ultravist; Bayer HealthCare Pharmaceuticals, Berlin, Germany) with 300 mg iodine/mL was injected at a flow rate of 4–6 mL/s, followed by an injection of 30–40 mL of saline solution. The scanning was delayed by 20, 60, 90, and 120 s for the arterial, portal vein, hepatic vein, and delayed phases, respectively. A 1.5-T MR scanner (Ingenia; Philips) or 3.0-T MR scanner (Discovery 750; GE Healthcare) with corresponding 8-channel abdominal phased-array coils were used for MRIs. Conventional axial spin-echo sequence T1-weighted images, axial fast spin-echo sequence with fat suppression T2-weighted images, and single excitation of spin-echo diffusion-weighted images were acquired. For multiphasic contrast-enhanced MR scanning, a 15-mL intravenous bolus of gadopentetate (Magnevist; Bayer HealthCare Pharmaceuticals) was injected into the elbow vein at a flow rate of 2.5 mL/s before axial and coronal T1 high-resolution isotropic volume excitation scanning. The contrast scan was delayed by 25, 80, 110, and 200 s for the arterial, portal, hepatic venous, and delayed phases, respectively. The Philips S2000 or Toshiba Aplio500 (Toshiba, Tokyo, Japan) systems were used for ultrasound imaging. The frequency of the convex array probe was 3.5–5.0 MHz.

### Image assessment

All images were retrospectively reviewed by two senior radiologists (with 15 and 20 years of experience, respectively) who were blinded to the patients’ clinical information until a consensus was reached. The radiological findings were analyzed considering the following criteria: location of lesion; number of lesions; shape; size (maximum diameters on axial, coronal, or sagittal images); tumor density/intensity on unenhanced CT and MRI images (the signals of the tumor-containing organ were defined as isodense and isointense, respectively); contrast enhancement pattern (signal intensities or densities similar to or higher than those of adjacent organs were defined as mildly or significantly enhanced); presence of a “halo sign” (a thin hyperintense ring surrounding the tumor on T2-weighted images); and presence of pseudocapsules (a thin hypointense ring surrounding the tumor on T2-weighted images;).

Ultrasound examinations were performed at two hospitals by senior ultrasound physicians with 16 years of experience each. The images were retrospectively analyzed by another senior ultrasound physician with 25 years of experience. The physicians agreed on the following signs: tumor location, size, shape, boundary, internal echo, relationship with adjacent organs, color Doppler blood flow, and enhancement intensity and mode of the tumor on contrast-enhanced ultrasound.

### Pathological examination

In all 13 cases, the tumors were resected, and the tissue samples were examined using hematoxylin and eosin staining as well as immunohistochemical EnVision staining. The antibodies that were used are listed in Table 1. One pathologist with 13 years of experience analyzed the histopathologic specimens after surgery. Appropriate positive and negative controls were evaluated simultaneously. In situ hybridization for Epstein–Barr virus (EBV)-encoded RNA (EBER) was performed in the 13 cases using an EBV probe in situ hybridization kit (Novocastra, Newcastle upon Tyne, United Kingdom). The manufacturer’s instructions were followed with no modification, and a known positive control was used to ascertain the sensitivity of the assay.

**Table 1:** Antibodies used for immunostaining

Antibodies	Source	Clonality	Dilution	pretreatment
CD 21	Dako	Monoclonal	1:50	HT
CD 35	Dako	Monoclonal	1:25	PC
CD 23	Dako	Monoclonal	1:50	PC
ALK 1	Novocastra	Monoclonal	1:25	HT
CD 30	Dako	Monoclonal	1:20	PC
EMA	Dako	Monoclonal	1:50	HT
CD 68	Dako	Monoclonal	1:20	TP
S-100 pretein	Dako	Monoclonal	1:800	HT
SMA	Dako	Polyclonal	1:500	No pretreatment
desmin	Dako	Polyclonal	1:50	No pretreatment
CD 1a	Novocastra	Monoclonal	1:20	HT
CD 3	Dako	Monoclonal	1:800	PC
CD20	Dako	Monoclonal	1:1600	HT

*HT* heating at 95 °C in citrate buffer(10 mmol/L, PH 6.0) for 30 min, *PC* pressure cooking in EDTA for 2 min, *TP* trypsinization with 0.1% trypsin for 20 min at room temperature

### Statistical analysis

Statistical analyses were performed using SPSS (version 20.0, IBM, New York, USA). A Student’s t-test was used to compare the patients’ age at onset and the maximum tumor diameters in the liver and spleen. The Chi-square test was used for qualitative variables, such as sex, the presence or absence of symptoms, density, signal intensity, enhancement pattern, the presence or absence of pseudocapsules, and “halo signs.” P values < 0.05 were considered statistically significant.

## Results

### Clinical characteristics

Our patient cohort included seven men and six women aged 36–88 years. Three patients presented with abdominal distension and epigastric pain, and two presented with low-level fever. The other eight asymptomatic cases were detected during health checkups. In these eight cases, physical examinations revealed no signs of tenderness or rebound tenderness. Only one case had an increased white blood cell count before surgery. The white blood cell count and C-reactive protein levels were high at 19.5 × 10^9^/L and 31.1 mg/L, respectively; whereas alpha-fetoprotein, carcinoembryonic antigen, carbohydrate antigen 125, and carbohydrate antigen 19–9 were within the normal ranges. Both hepatitis B virus surface antigen and the antibody were negative. All tumors were resected without radiological recurrence during the follow-up periods of 10–24 months.

The clinical manifestations of IPT-like FDCS and comparisons of the liver and spleen lesions were listed in Tables 2 and 3. No statistically significant differences in sex, clinical manifestation, median age, or the number of nodules were observed.

**Table 2:** Clinical manifestations of inflammatory pseudotumor-like follicular dendritic cell sarcomas of the liver and spleen

	Liver (n = 2)	Spleen (n = 9)		Liver and spleen (n = 2)	P value
Sex					0.706
Male	2		4	1	
Female	0		5	1	
Clinical manifestation					0.119
Fever or abdominal discomfort	1		2	2	
Physical examination	1		7	0	
Median age (years)	67 (53–81)		59 (36–88)	56 (46–66)	0.983
Number of nodules					0.052
Single	1		8	0	
Multiple	1		1	2

**Table 3:** Clinical characteristics of inflammatory pseudotumor-like follicular dendritic cell sarcomas of the liver and spleen

Case	Age (years)	Sex	Location	Symptoms	Treatment	Follow-up (months)	Examination method
1	81	Male	Liver	No	Excision	12	CT and MRI
2	53	Male	Liver	Abdominal distension	Excision	24	MRI, ultrasound
3	76	Female	Spleen	No	Excision	10	MRI
4	49	Female	Spleen	No	Excision	24	CT and MRI
5	73	Male	Spleen	No	Excision	18	CT, ultrasound
6	66	Female	Liver and spleen	Epigastric pain	Excision	20	CT, ultrasound
7	55	Male	Spleen	No	Excision	20	CT and MRI
8	46	Male	Liver and spleen	Fever	Excision	24	CT and MRI ultrasound
9	62	Female	Spleen	No	Excision	18	MRI
10	43	Female	Spleen	Abdominal distension	Excision	24	CT and MRI
11	36	Male	Spleen	Fever	Excision	24	CT and MRI
12	41	Female	Spleen	No	Excision	17	MRI
13	88	Male	Spleen	No	Excision	12	MRI

*CT* computed tomography, *MRI* magnetic resonance imaging

### Multi-detector computed tomography findings

**Fig. 1:**
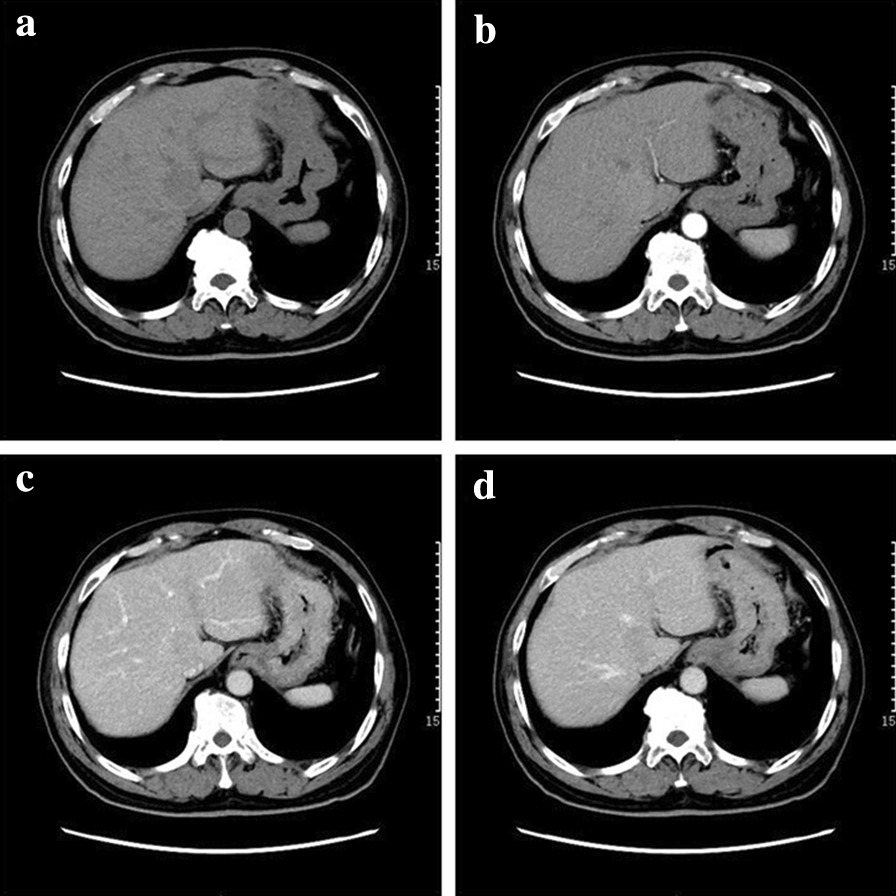
A 64-year-old male patient with inflammatory pseudotumor-like follicular dendritic cell sarcoma of the liver. **a** Plain computed tomography scan showing a round, slightly hypodense lesion with homogeneous density and no obvious necrosis, calcification, or bleeding in the caudate lobe of the liver. **b** Slight enhancement in the arterial phase. **c** and **d** Decreased enhancement in the portal and delayed phases, respectively

**Fig. 2:**
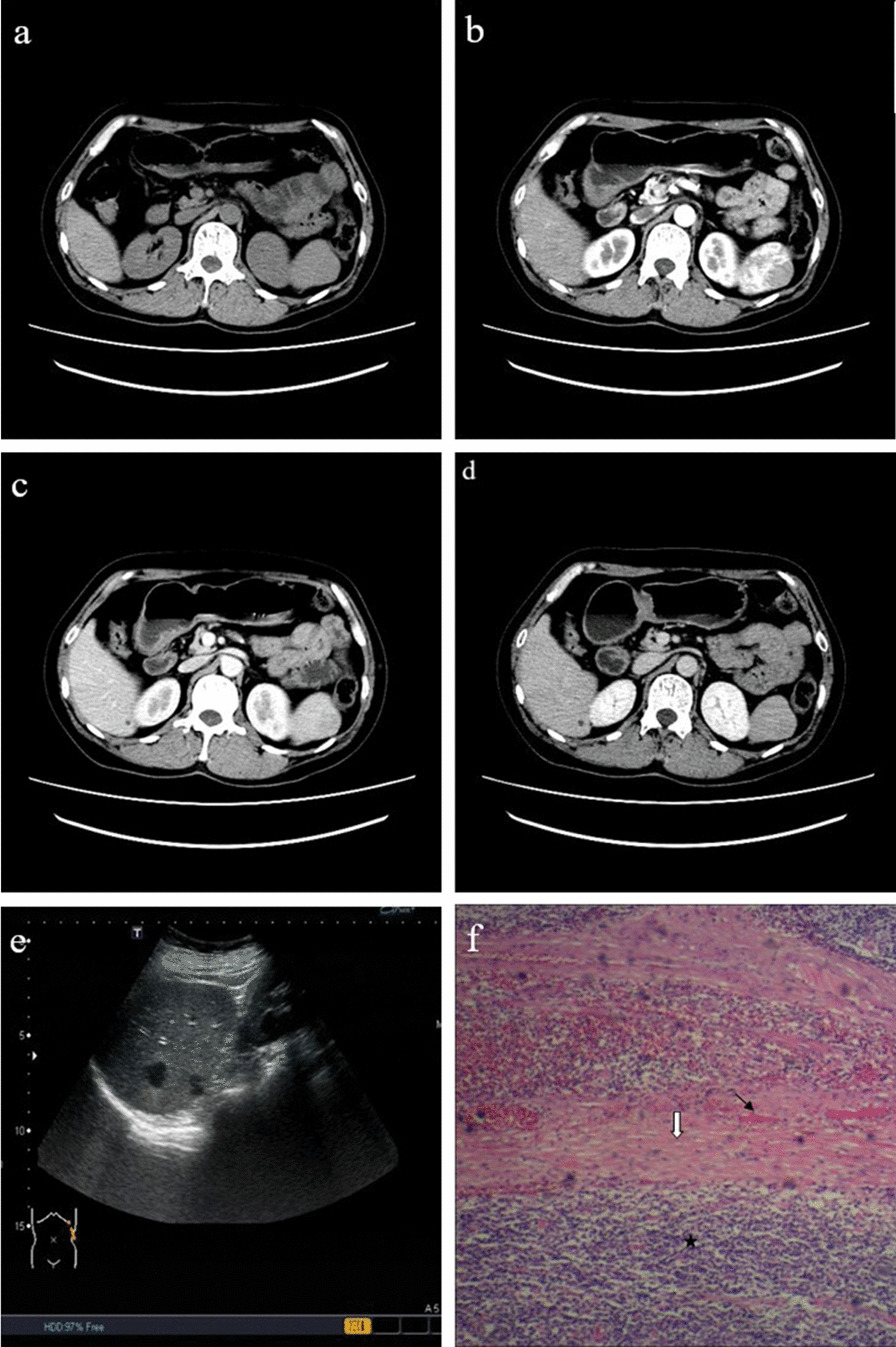
A 45-year-old male patient with inflammatory pseudotumor-like follicular dendritic cell sarcoma of the spleen. **a** Plain computed tomography scan showing a round slightly hypodense lesion of the spleen. There was homogeneous density and, no obvious signs of necrosis, calcification, or bleeding. **b** and **c** After contrast enhancement, the relatively hypodense lesion is observed in all phases. **d** Circular enhancement at the lesion’s edge in the delayed phase. **e** Ultrasound image, revealing a round-like lesion with a clear boundary and homogeneous hypoechogenicity in the spleen. **f** Pathological examination of the lesion (hematoxylin and eosin [HE], ×100). A large number of inflammatory cells infiltrate the tumor (black star). The formation of a fibrous pseudocapsule (white arrow) with blood vessels (black arrow) in the fibrous capsule can be seen at the edge of the tumor

**Fig. 3:**
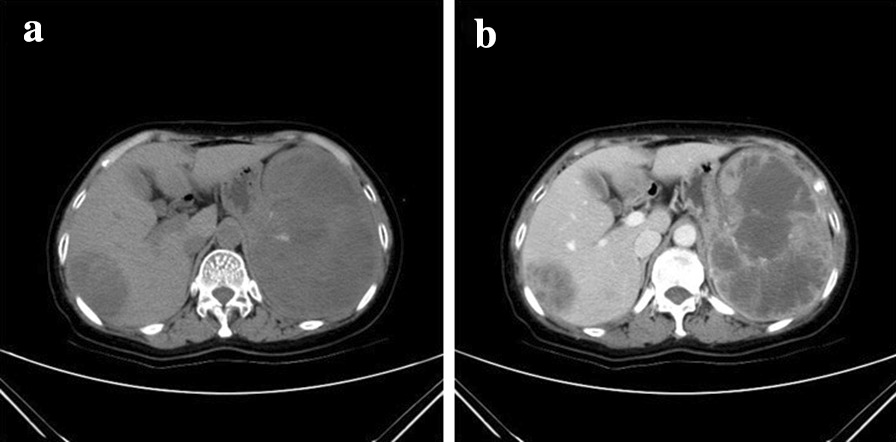
A 57-year-old female patient with multiple inflammatory pseudotumor-like follicular dendritic cell sarcomas in the liver and spleen. **a** The plain computed tomography scan reveals round-like hypodense masses with uneven densities in the spleen and liver. **b** After contrast enhancement, the masses show a honeycomb structure, and no enhancement can be seen in the central necrotic area

The imaging characteristics of IPT-like FDCS differed slightly between the liver and spleen (Table 4). Most unenhanced CTs showed a circular or quasi-round, slightly hypodense mass with a clear boundary. The tumor density was homogeneous in five cases. Obvious necrosis was found in three cases, and no calcification or bleeding was detected. The tumors in the liver were slightly enhanced in the arterial phase, decreased in the portal and delayed phases, and showed slightly lower densities than the liver parenchyma (Fig. 1). Splenic tumors showed mild continuous enhancement in five cases. Because the tumor enhancement was lower than that of the parenchyma, the lesions were always hypodense, and annular enhancement was seen in the delayed phase (Fig. 2a–d). In the other two cases, the tumors in the spleen were significantly enhanced, and one of them showed honeycomb changes. The solid region showed heterogeneous enhancement after contrast administration, sparing the central necrotic region (Fig. 3).

**Table 4:** Imaging characteristics of inflammatory pseudotumor-like follicular dendritic cell sarcomas in the liver and spleen

	Liver	Spleen	P value
Diameter (cm)	3.6 ± 1.1	5.2 ± 2.6	0.12
Density			1.0
Iso	0 (0%)	1 (14%)	
Hypo	3 (100%)	6 (86%)	
T1 signal intensity			0.045*
Hypo	3 (100%)	2 (22%)	
Hyper	0 (0%)	7 (78%)	
T2 signal intensity			0.045*
Hypo	0 (0%)	7 (78%)	
Hyper	3 (100%)	2 (22%)	
Enhancement pattern			0.033*
Significantly enhanced	3	1	
Mildly enhanced	1	10	
Pseudocapsule	2/3 (66%)	7/9 (78%)	0.637
Necrosis	1/4 (25%)	4/11 (36%)	0.462
“Halo sign”	2/3 (67%)	0/9 (0%)	0.045*

*p < 0.05

### MRI findings

**Fig. 4:**
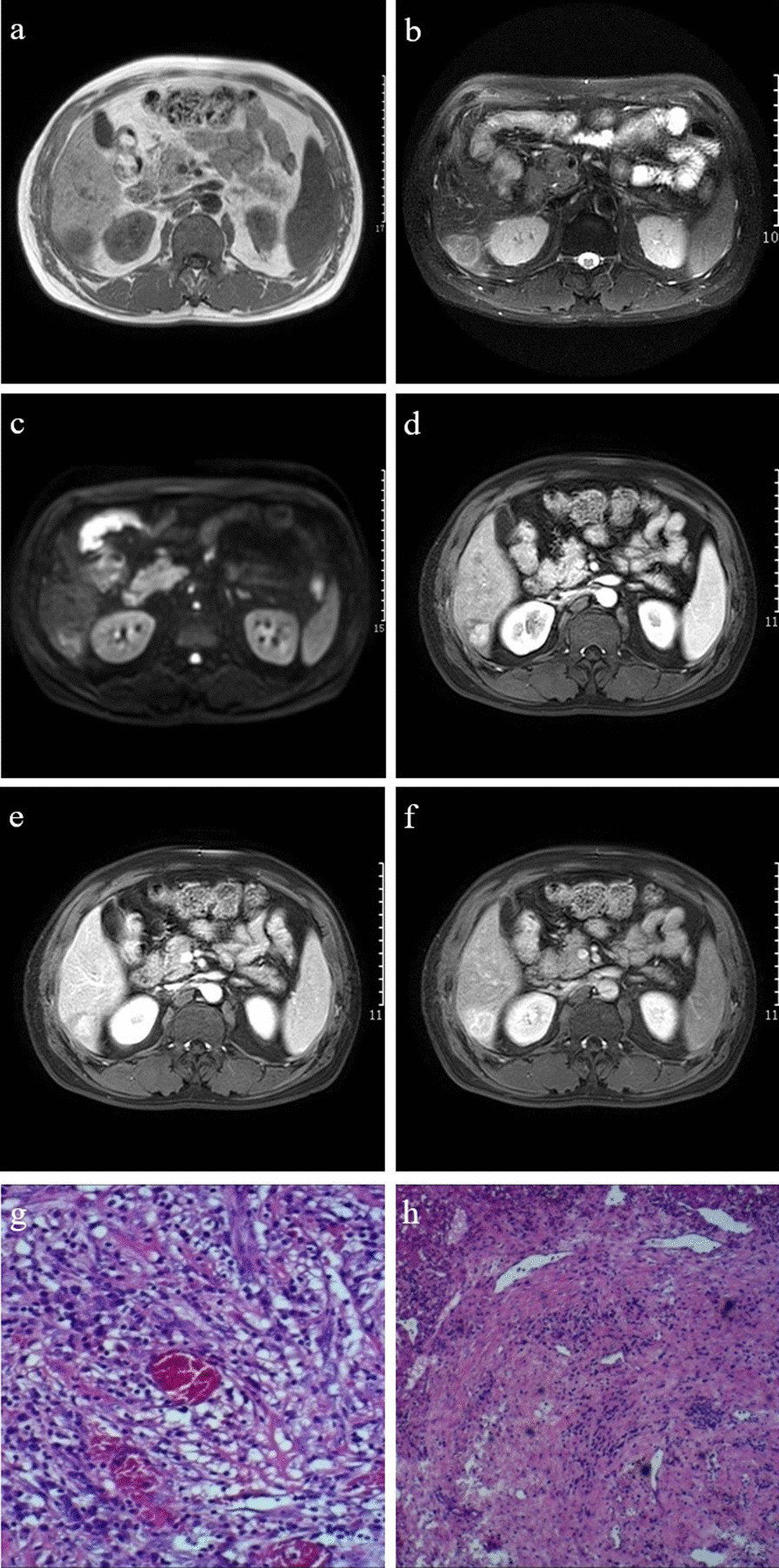
A 46-year-old male patient with inflammatory pseudotumor (IPT)-like follicular dendritic cell sarcoma (FDCS) in the liver. **a** The lesion shows a slightly lower signal intensity in the T1-weighted image sequence, and the boundary is not clear. **b** On the T2-weighted image sequence, the lesion is slightly hyperintense, and a “halo sign” can be seen at the edge of the lesion. **c** The lesion shows heterogeneous hyperintense signals on the diffusion-weighted imaging sequence. **d** The lesion shows an obvious central nodular enhancement in the portal vein phase. **e** Progressive enhancement in the venous phase with relatively low enhancement at the lesion’s margin. **f** The enhancement of the lesion decreases in the delayed phase and a rim enhancement can be seen at the edge. **g** IPT-like FDCS of the liver, demonstrating abundant infiltration of inflammatory cells and the distribution of capillaries (hematoxylin and eosin [HE], ×200). **h** Small vessels can also be seen in the fibrous pseudocapsule of the tumor (black star; HE, ×100)

**Fig. 5:**
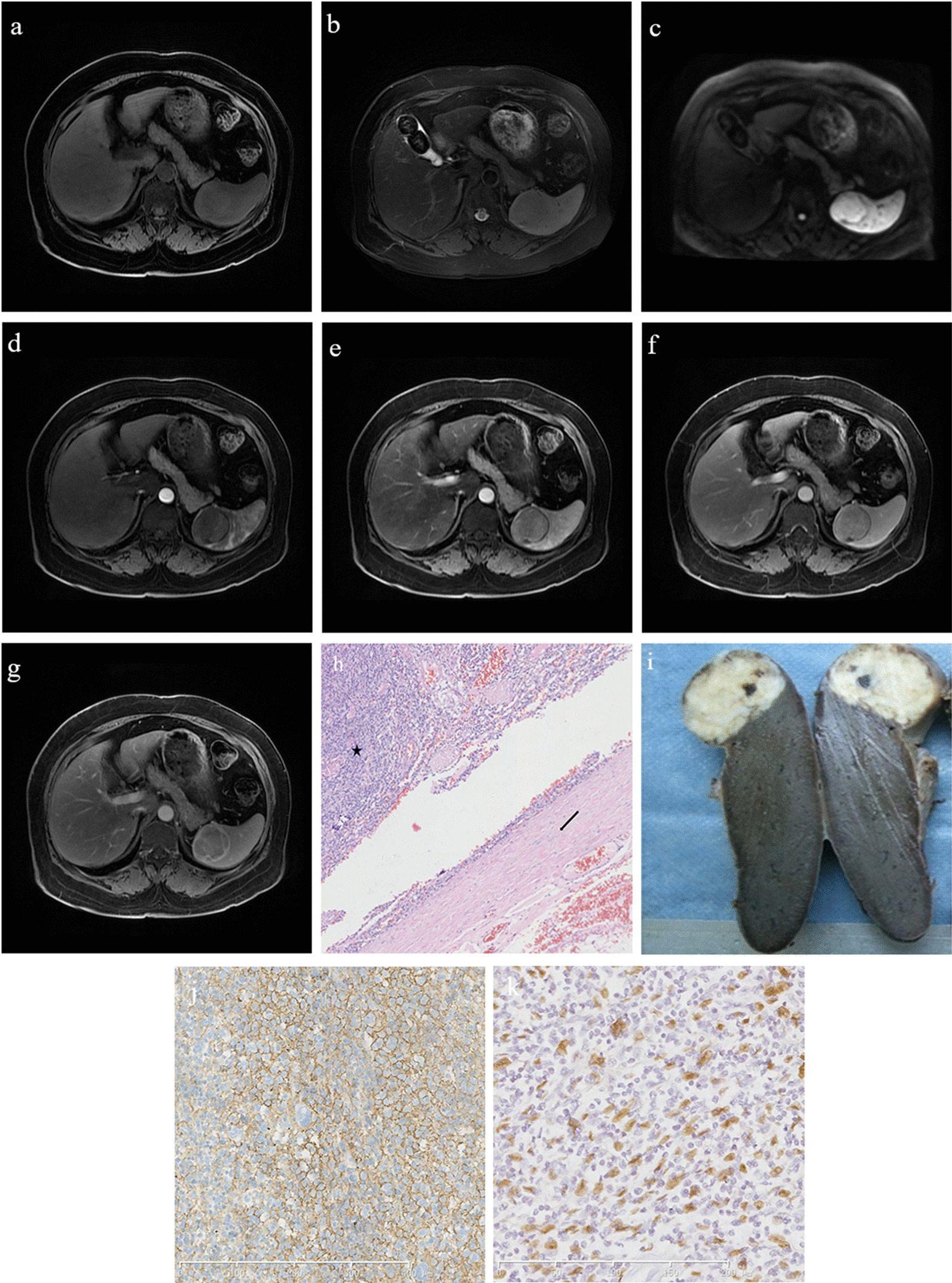
A 37-year-old male patient with inflammatory pseudotumor-like follicular dendritic cell sarcoma of the spleen. **a** The mass shows a slight hyperintensity on the T1-weighted image sequence. **b** On the T2-weighted image sequence, the tumor shows a slightly hypointense signal, and its edge displays a hypointense annular signal representing the pseudocapsule. **c** The mass shows a slightly hyperintense signal on diffusion-weighted imaging. **d**–**g** The contrast enhancement of the mass is mild in each phase, and the pseudocapsule shows delayed enhancement. **h** On histopathological examination, the tumor (black star) is surrounded by dense fibrous tissue (black arrow; hematoxylin and eosin, ×100). **i** Macroscopic examination showing a grayish-white tumor with uniform texture and clear boundaries. **j** CD21 is diffusely positive. **k** Epstein–Barr virus is detected in almost all of the tumor cells with positive dark staining of their nuclei

All liver tumors demonstrated slightly hypointense signals on T1-weighted images (Fig. 4a) and slightly hyperintense signals on T2-weighted images, with unclear edges and a “halo sign” present in 2 of 3 cases (67%) (Fig. 4b). The diffusion-weighted imaging sequence showed slightly high or high signal intensities (Fig. 4c). The liver lesions demonstrated substantial enhancements from the center to the periphery in the arterial phase (Fig. 4d). The enhancement amplitude of the lesions in the portal, venous, and delayed phases tended to be homogeneous and decreased in varying degrees, and annular enhancements could be seen (Fig. 4e, f). Seven of the nine cases with splenic lesions showed slight hyperintensities on T1-weighted images (Fig. 5a), slight hypointensities on T2-weighted images (Fig. 5b), slightly high or high signal intensities on diffusion-weighted images (Fig. 5c); and a hypointense ring on T2-weighted images could be seen at the lesion’s margin (Fig. 5b). There was mild-to-moderate heterogeneous enhancement; after enhancement, the amplitude was always lower than that of the normal splenic tissue, and annular enhancement could also be seen in the delayed phase (Fig. 5d–g). Significant differences between liver and spleen IPT-like FDCSs were found when comparing T1 and T2 signal intensities, enhancement patterns, and the presence of “halo signs” (Table 4).

### Ultrasonographic findings

Two-dimensional ultrasound imaging demonstrated round-like lesions inside the spleen, with clear boundaries in two cases (Fig. 2e), unclear boundaries in the other two cases, and homogeneous and heterogeneous hypoechogenicity in two cases each. Patchy, slightly hyperechoic, small dark areas could be seen in one case, and a small dark area could be seen in another case. Strip or short-rod vascular signals could be seen in tumors with a pulse wave registered in the center and at the margins on color Doppler, and their velocity ranged from 90 to 140 cm/s. Only one case underwent contrast-enhanced ultrasound imaging and showed slight and rapid heterogeneous enhancement from the periphery to the center. Not only did the enhancement appear slightly earlier in the tumor than in the surrounding tissue, but it disappeared earlier as well. The number of cases examined by ultrasound was insufficient for statistical analyses.

### Pathology results

The pathological feature of IPT-like FDCS in both the liver and spleen was very similar. Macroscopically, masses were round or nodular with diameters ranging from 3.5 to 12.0 cm (average, 6.5 cm). Sections were solid and grayish-white with clear boundaries, and fibrous pseudocapsules could be seen (Fig. 5i) pushing into the surrounding tissue. Necrosis was detected in the center of the larger lesions in three cases. Histologically, presence of fibrous pseudocapsule with vitreous degeneration was confirmed (Fig. 5h). The neoplastic cells were dispersed or arranged in vague fascicles within a prominent lymphoplasmacytic infiltrate. The tumor cells showed a spindled shape with an unclear boundary and rich reddish cytoplasm. The nuclei were elongated and vesicular with small but distinct nucleoli. Mitosis was race, and multinucleated giant cells were seen in some cases. IPT-like FDCS in the liver had a rich distribution of small blood vessels and capillary networks (Fig. 4g, h). The vascular distribution could also be seen in the fibrous capsule of some cases (Fig. 2f).

Immunophenotypically, the tumor cells were strongly positive for CD21 (Fig. 5j) and CD35 in 13 cases and for CD23 in three cases. Clusterin staining showed strong positive findings in the cytoplasm in three cases. Some tumor cells were positive for CD68, smooth muscle actin, vimentin and epidermal growth factor receptor. The Ki-67 index was 5–30%. All cases were positive for EBER by in situ hybridization analysis (Fig. 5k).

### Treatment outcomes and follow-up

All 13 patients underwent surgical excision without adjuvant chemotherapy. The tumors were resected, and splenectomy and/or right or left hepatectomy and wedge resection of the liver were performed, accordingly. All cases were followed up postoperatively for periods ranging from 10 months to two years, and none exhibited local recurrence or distant metastasis.

## Discussion

IPT-like FDCS is more common in adults. Compared with classical FDCS, IPT-like FDCS is more frequently observed in female patients; however, there was no statistically significant difference in sex in our study. Most patients had no clinical symptoms or those that were unspecific. In our study population, only two patients had prodromal symptoms of low-level fever, and three patients had symptoms of abdominal distension and pain, whereas eight patients had no obvious typical symptoms. This is consistent with previously reported findings [7]. While IPT-like FDCS is very similar in morphology to IPTs, it has the same cytological morphology and immunophenotype as classical FDCS; that is, the tumor cells are scattered in an inflammatory background, including lymphocytes and plasma cells. In our study, IPT-like FDCS tumor cells expressed CD21 and CD35, while partly expressing CD68, smooth muscle actin, vimentin, and clusterin. Studies have shown that almost all cases of IPT-like FDCS are associated with the Epstein–Barr virus [6–9]. All patients in our cohort were positive for CD21, CD35, and the Epstein–Barr encoding region.

FDCS has a low degree of invasion but high local recurrence and metastasis rates, making it a moderately malignant tumor. IPT-like FDCS is more inert, and surgical resection is the first choice of treatment. In patients with tumor recurrence or those who cannot be treated by surgery, chemotherapy or radiotherapy can be administered [10]. None of the 13 patients showed tumor recurrence at follow-up, which is similar to reports from previous studies [11].

To date, most imaging findings reported have been from individual cases or small cohorts; large-scale data of imaging findings are scarce. To our knowledge, this study represents the largest radiologic-pathologic correlation study of IPT-like FDCS cases. Imaging findings of IPT-like FDCS in the liver and spleen revealed both similarities and differences. In both intrahepatic and intrasplenic tumors, the density was often homogeneous and hypodense on CT. We speculate that slow tumor growth, sparse tumor cells, and abundant blood supply may be the reasons behind this observation. Seven of eight cases showed slight hypodensities on CT; only three tumors (> 5 cm in diameter) had a central hypodense necrotic area. After contrast medium injection and enhancement of the solid components, the enhancement of IPT-like FDCS in the spleen was found to be weaker than that in the liver, which may be related to the obvious enhancement of the spleen itself. On MRI scans, IPT-like FDCS was more characteristic. Intrasplenic IPT-like FDCS showed relative hypointensities on T2-weighted images due to the hyperintense signals on T2-weighted images of the spleen itself. Our study confirmed that the pseudocapsule formed at the tumor boundaries in nine cases presented as hypointense signals on the T2-weighted image sequence, because it mainly comprised collagen fibers, which was consistent with other studies [12]. Our study further found that the enhancement of the tumor pseudocapsule in the delayed phase was due to the blood vessel distribution in the pseudocapsule of the tumor. IPT-like FDCS exhibits slightly hyperintense or hyperintense signals on diffusion-weighted imaging, which may be closely related to the higher cell density. The spleen is rich in blood sinuses, which may cause an obvious T2 shine-through effect; the spleen parenchyma has hyperintense signals on diffusion-weighted imaging, whereas the IPT-like FDCS signal in the spleen is relatively lower. The mild-to-moderate contrast enhancement of tumors in the liver may be related to the abundance of micro-vessels and inflammatory cells. The “halo sign” could only be seen in a certain proportion of IPT-like FDCSs of the liver, which is consistent with the incomplete tumor capsule and the infiltration of plasma cells and lymphocytes in the peritumoral edema, as seen under the microscope. IPT-like FDCSs of the spleen displayed homogeneous or heterogeneous hypoechoic lesions, consistent with ultrasonographic findings in other studies [3]. The patchy hyperechoic area detected in the tumor was related to the infiltration of numerous inflammatory cells [13, 14]. The abundant strip or short-rod blood flow signals in the periphery, and the circular enhancement around lesions in contrast-enhanced ultrasonography, were consistent with the blood vessel distribution in the pseudocapsule of the tumor in the pathological findings. Therefore, the imaging findings of IPT-like FDCS in the liver and spleen have their own specific characteristics and need to be treated differently in terms of diagnosis.

Although the imaging manifestations of the tumors vary, IPT-like FDCS contrast enhancement can be divided into three types according to the enhancement characteristics, and this is closely related to the tumor’s histopathological features. (a) Centrifugal enhancement type: following contrast agent administration, the center of the tumor was substantially enhanced in the arterial phase, further enhanced in the portal and venous phases, the scope of the enhancement was enlarged, and the edge of the tumor was enhanced in the delayed phase. In our study, this type was only found in the liver (Fig. 4). (b) Marginal enhancement type: the boundary of this type was clear on a plain scan, with uniform density or signal intensity on CT and MRI, respectively. Mild enhancement of a thin ring in the arterial and portal phases, followed by continuous enhancement in the delayed phase, was seen. Pathologically, the center of this type lacked blood supply. This tumor type contained many collagen fibers, and a pseudocapsule was sometimes formed by fibrous tissue and infiltrating inflammatory cells (Fig. 5). (c) Grid enhancement type: on plain CT scan, honeycomb-like hypodensities were visible in tumors with a clear boundary. Multiple areas of hypointensities on T1-weighted images and hyperintensities on T2-weighted images were observed inside the tumor, with an isointense septum. Obvious continuous enhancement changes were observed around the tumor and its internal septum. Hypodense areas on CT indicated necrosis, whereas iso- or slightly hyperdense areas indicated the proliferation of fibrous tissue. This tumor type was often larger, the capsule was incomplete, and its biological behavior suggested that it was more aggressive (Fig. 3).

In summary, IPT-like FDCS is a rare tumor that is mainly observed in the liver and spleen. By correlating imaging and pathology findings, our comparative study of 13 IPT-like FDCS cases identified characteristic imaging features of this type of tumor in the liver and spleen. Although these tumors are usually indolent, surgical resection is still the fundamental treatment and requires postoperative follow-up.

Our study has some limitations, with the relatively small sample size being the most important. IPT-like FDCS is an uncommon tumor; moreover, patients with IPT-like FDCS may never undergo diagnostic imaging because they are asymptomatic. Second, owing to the retrospective nature of this study, we did not obtain both multi-detector CT and MR images for the majority of patients. Additionally, different MRI scanners and techniques were used. However, these issues are unavoidable due to the rarity of this tumor type, and this limitation should not have significantly affected the radiological characteristics studied.

## Conclusions

We believe that complete CT and MRI examinations may be of great help in the diagnosis of IPT-like FDCS. Consideration should be given to a possible diagnosis of IPT-like FDCS when single or multiple masses are found with different patterns of enhancement on CT and MRI, a “halo sign,” the presence of a pseudocapsule appearance, and annular enhancement in the delayed phase are observed.

## Data Availability

The datasets used and/or analyzed during the current study are available from the corresponding author on reasonable request.

## Abbreviations

CT: Computer tomography
MRI: Magnetic resonance imaging
IPT-like FDCS: Inflammatory pseudotumor-like follicular dendritic cell sarcoma
